# Pre-corneal tear film thickness in humans measured with a novel technique

**Published:** 2011-03-22

**Authors:** Kaveh Azartash, Justin Kwan, Jerry R. Paugh, Andrew Loc Nguyen, James V. Jester, Enrico Gratton

**Affiliations:** 1 Biomedical Engineering, University of California Irvine, Irvine, CA; 2 Southern California College of Optometry, Fullerton, CA; 3 California State University, Fullerton, CA; 4 Gavin Herbert Eye Institute, University of California Irvine, Irvine, CA

## Abstract

**Purpose:**

The purpose of this work was to gather preliminary data in normals and dry eye subjects, using a new, non-invasive imaging platform to measure the thickness of pre-corneal tear film.

**Methods:**

Human subjects were screened for dry eye and classified as dry or normal. Tear film thickness over the inferior paracentral cornea was measured using laser illumination and a complementary metal–oxide–semiconductor (CMOS) camera. A previously developed mathematical model was used to calculate the thickness of the tear film by applying the principle of spatial auto-correlation function (ACF).

**Results:**

Mean tear film thickness values (±SD) were 3.05 μm (0.20) and 2.48 μm (0.32) on the initial visit for normals (n=18) and dry eye subjects (n=22), respectively, and were significantly different (p<0.001, 2-sample *t*-test). Repeatability was good between visit 1 and 2 for normals (intraclass correlation coefficient [ICC]=0.935) and dry eye subjects (ICC=0.950). Tear film thickness increased above baseline for the dry eye subjects following viscous drop instillation and remained significantly elevated for up to approximately 32 min (n=20; p<0.05 until 32 min; general linear mixed model and Dunnett’s tests).

**Conclusions:**

This technique for imaging the ocular surface appears to provide tear thickness values in agreement with other non-invasive methods. Moreover, the technique can differentiate between normal and dry eye patient types.

## Introduction

Study of the precorneal tear film has enjoyed renewed interest due to evidence that dry eye syndrome (DES) is a common ophthalmic condition, potentially affecting 5% to more than 30% of the adult population in the United States [1], and one that adversely impacts the quality of life of those who suffer from it [2]. One important trend in research has been the development of non-invasive methods to characterize the tear film. These include non-invasive stability measures [3,4], approaches to quantify lipid layer thickness [5] and behavior [6], and measurement of total tear film thickness [7]. Tear film thickness in particular offers a quantitative approach to characterize DES, wherein the thickness is presumably less than under normal conditions due to tear compositional or physiologic deficiencies leading to instability and excessive evaporation. Moreover, tear film thickness measurement offers an objective approach to monitor the effect of dry eye treatment. An abnormally thin tear film is of significance because it may possibly reflect a state of hyperosmolarity of the ocular surface, which is thought to cause damage and result in many of the dry eye symptoms [8].

King-Smith et al. [9] used interferometry and by evaluating the reflectance spectra from the ocular surface, were able to noninvasively quantify human tear film thickness. They obtained measurements of approximately 3.0 µm for the normal human tear film [9]. While scientifically sound, the reflectance spectra method is at present available in very few laboratories, is technically complex and requires relatively expensive equipment.

Another non-invasive method that has been applied to tear film thickness measurement is optical coherence tomography (OCT) [10]. OCT is an optical signal acquisition and processing method that can be considered an interferometric technique. OCT was first used to asses tear film by Wang et al. [11,12] reporting values of approximately 3 µm, similar to those of King-Smith et al. [7,9,13,14] While non-invasive, a concern relative to OCT is that the thickness determination is indirect rather than direct measurement [12].

In the current work a novel technique was applied to quantify the thickness of the pre-corneal tear film in humans; termed: Fluctuation Analysis by Spatial Image Correlation (FASIC). Thus far, the method has demonstrated efficacy in measurement of tear film thickness in animal models [15] providing thickness values in agreement with prior measurements. The system is robust, portable, low-cost (the relative cost of this technique in its current configuration is under $1,000.00.), and easy to operate. This technique involves the quantitative assessment of periodic interference patterns in a series of images, from which the ocular tear film thickness can be calculated using a mathematical model that translates the spatial fluctuations into thickness information.

The aim of the investigation presented in this article was to measure the thickness of the human tear film using FASIC. This was a study to generate preliminary tear film thickness data from normal (i.e., non dry eye) and dry eye subjects in a clinic-based population. In addition, we examined the short-term (i.e., one week) repeatability of the method in a single setting and the temporal effect of a viscous lubricating eye drop on tear film thickness.

## Methods

### Instrumentation and the FASIC technique

**Figure 1:**
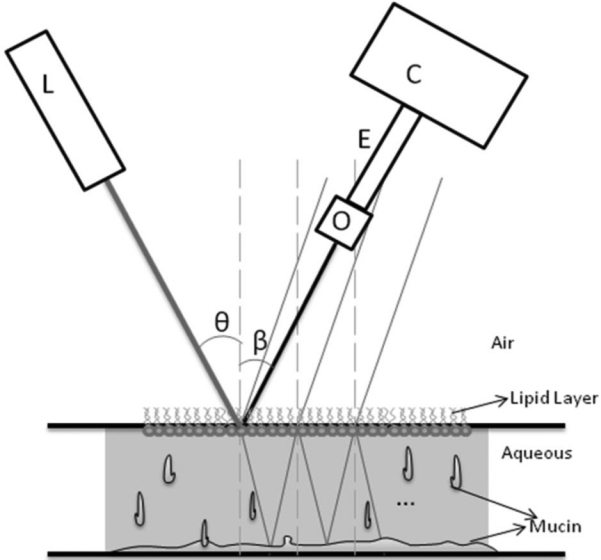
An schematic of the physical principles and the components in FASIC setup. The low-power, 635 CW laser (L) illuminates the ocular surface at an incident angle of θ. The cMOS camera (C) is connected to a 1× objective lens (O) through an extender tube (E) captures the images at an observation angle of β.

A schematic of the FASIC setup is shown in Figure 1. A 635 nm diode laser (LXC6351AH; Lasermate, Pomona, CA), with less than 1mw of power (0.7 mW–0.9 mW), was used as a monochromatic coherent light source. The wavelength and power of this light source were within the standards of Laser Institute of America for eye safety [16]. The laser beam was collimated onto the inferior cornea for all subjects, approximately mid-way between the visual axis and the limbus at along the vertical meridian and had a diameter of approximately 2 mm. Therefore the measurements represent an average tear film thickness across the illumination area. The incident angle, which plays an important role in obtaining the true thickness value, was approximately 12 degrees. The observation angle was slightly greater than the incident angle in this apparatus. The scattered and reflected light was focused back to a cMOS camera (PL-A662-KIT; Pixelink, Ottawa, ON, Canada) through an objective lens (Mitutoyo Compact CF 1× Objective; Edmund Optics, Barrington, NJ). The objective was coupled to the complementary metal–oxide–semiconductor (CMOS) camera through an extender tube having a length of 15 cm. To capture an image, the camera assembly was focused 68 mm from the cornea. The camera assembly and the light source were mounted onto an X-Y-Z translational stage for backward/forward, up/down and sideways movements. A stack of images (256×256 pixels each) was streamed directly to a computer from the cMOS camera through a Firewire cable with the exposure time set at 1 ms. The image size was chosen such that it covers the 2 mm illuminating area on the eye. It also allows for capturing images at a fast frame rate. This high frame rate allowed capturing of fluctuations in the ocular surface at about 300 Hz and to compensate for motion artifacts. For most of the experiments 1,000 frames were collected for a total acquisition time of approximately 4 s. The data acquisition started approximately 1.5 s after the subject blinked. For the thickness analysis, the first 500 frames were analyzed. This means that only the thickness data from 1 to 2 s following eye opening were measured, and the data that were analyzed belonged to 3 s following a blink; less than the average inter-blink interval. For subjects with approximately 3 s tear break-up time (TBUT), the data were analyzed such that the FASIC measurement does not go over the TBUT.

Prior to the study, the system was calibrated using parylene films fabricated on a silicon substrate. The thickness obtained with the FASIC approach measured film thickness accurately compared against profilometer measurements. Using these films in the range of tear film thickness the FASIC method demonstrated a coefficient of variation (COV) of approximately of 0.20 to 0.35% (4.92 and 2.90 μm thickness standards, respectively). In the human tear film, the COV was much greater, ranging from about 6.7 to 13.1% for the non-dry eye and dry eye subjects, respectively (visit 1). However, this relatively small variability allowed meaningful differences to be uncovered under the several test conditions.

### Spatial autocorrelation and physical principles

**Figure 2:**
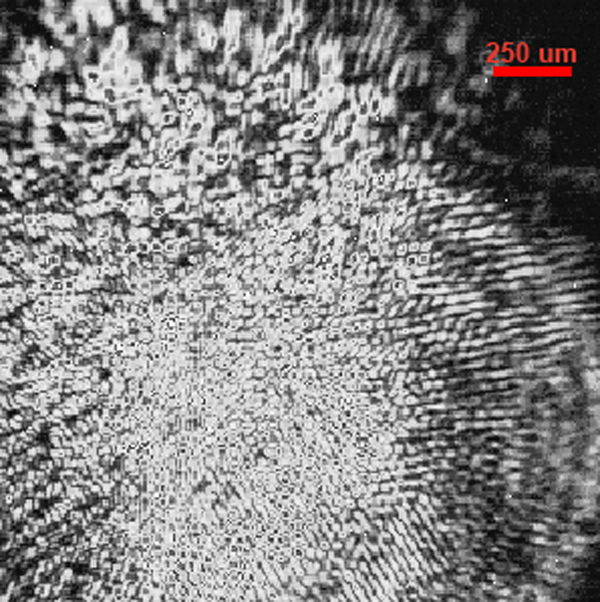
This figure shows the raw camera image. In this image multiple interference patterns are apparent with different spacing, sizes and orientations. The period of these fringes is extracted by applying the spatial autocorrelation analysis and used in calculating the thickness of the tear film.

Spatial autocorrelation is a mathematical operation that measures and evaluates the degree of dependency of a set of data with itself. Petersen et al. [17] pioneered the application of this statistical analysis to the imaging and microscopy community. The spatial autocorrelation relationship is given by [17]:

$$G_{s}(\xi ,\psi )=\frac{{\langle I(x,y)I(x+\xi ,y+\psi )\rangle }_{x,y}}{{\langle I(x,y)\rangle }_{x,y}^{2}}-1$$

where *I(x,y)* represent the image intensity, ξ and ψ are the spatial increments in the x and y directions, respectively, and the angle bracket indicates the average over all the spatial locations in both x and y directions. The principles of autocorrelation analysis have been widely accepted in the imaging and microscopy community [18-21]. A raw camera image is shown in Figure 2. This figure illustrates various families of periodic fringes with different spacing and orientation. The features in the image are difficult to be discriminated by the human eye.

The periodic pattern that is observed at the ocular surface, originates from interference within the tear film and from scattering at the surface of the tear film. Existence of convoluted features such as lines, dots and other structures makes the camera image in general complex, although it follows the basic dependency on tear film thickness. These complex features are not picked up by simple inspection of the images and therefore, the pattern is only analyzed in the autocorrelation function and after selecting the correct periodic pattern. The factors affecting the periodic part of the spatial autocorrelation function have been experimentally determined and verified to depend on the inverse of the thickness of the film. In a previous work [15], it was shown that the thickness then can be calculated by:

$$t=\frac{K}{nP\sin(\theta )}$$

where *n* is the refractive index of the medium which is assumed to be 1.336 [22], and *θ* is the incident angle and *K* is a constant and was empirically obtained and includes the wavelength of the light as well. *P* is the period of the fringes available in the raw camera data which is calculated using the auto-correlation function (ACF).

### Human tear thickness studies

#### Study population

Normal (non-dry eye) and dry eye subjects were recruited by word of mouth and sourced from the existing dry eye database of the Southern California College of Optometry (Fullerton, CA). Thus, this represented a clinic-based sample. Subjects were designated as not having dry eye (normal) or having dry eye based on symptoms, tear stability, and corneal and/or conjunctival staining (see below). All subjects were 18 years of age or older. Major inclusion criteria included the ability to return for the repeatability visit within 7±2 days and willingness to discontinue soft contact lens wear or the use of topical drops 2 days before each measurement visit. Soft contact lens wearers were included, although it is not known whether lens wear affects tear thickness.

Major exclusion criteria included ocular surgery within the previous six months, evidence of active ocular infection, use of topical ocular medications other than artificial tears, rigid gas permeable contact lens wearers, and individuals with punctual plugs. Normals, and dry eye subjects who were mild, moderate, or severe were enrolled. Severity was assigned based on the worst sector corneal fluorescein staining grade of any of the 10 sectors for either eye according to the Efron scale [23] during the eligibility visit.

All subjects enrolled in the study were advised not to use topical eyedrops two days before each thickness determination (initial and repeatability visit) and contact lens wearers were advised to discontinue lens wear for two days before tear thickness determinations.

With the exception of the prohibition of pre-visit topical eyedrops, the subjects were assessed concurrent with existing dry eye management, which could have included omega 3 supplementation or use of the tetracycline drugs for meibomian gland dysfunction.

### Study procedures

Informed, written consent was obtained before initiating any study procedures. This study adhered to the tenets of the Declaration of Helsinki and was assigned clinical trial number NCT01014780.

Each subject had 3 visits to the clinic: an initial eligibility and characterization visit, and two quantitative tear thickness measurement visits with FASIC. The major experiments were tear thickness repeatability in normals and dry eye subjects, and a retention of effect study in the dry eye subjects using a viscous topical formulation (see below for details). All subjects were asked to return for a repeatability visit within one week (7 days), ±2 days. Since there is diurnal variation in several tear parameters, including stability [24,25], we re-measured tear thickness within the same one-half day (i.e., either morning or afternoon) as the initial assessment.

The subjects were classified as normal or having dry eye at the eligibility visit using a battery of tests. A medical history was taken, and dry eye symptoms were determined using the Schein questionnaire. Although there are several dry eye instruments available, we prefer the Schein survey since it is rapid (taking about 1 min to answer and score), was validated originally [26] and more recently in the modified form [27] against clinical diagnosis and has demonstrated repeatability in a treatment trial [28]. The original Schein questionnaire [26] was modified by adding a category of “never,” and assigning numerical values to each category (i.e., never=0; rarely=1, sometimes=2, often=3, all of the time=4). This allowed use of a semi-continuous numerical scale (0–24), for which a cut-point of 7 or greater for dry eye has been established [27].

Fluorescein tear breakup time (TBUT) was undertaken using liquid fluorescein (5.0 μl of 2.0% instilled using a micropipette) as part of the sequence used by Pflugfelder et al. [29] to assess TBUT, cornea staining and tear clearance. Although a historical breakpoint for fluorescein BUT has been <10 s [3,30] this criterion was reduced to <7 s by us for two major reasons.

First, the older studies used larger volumes of liquid fluorescein [31,32] and it has been demonstrated that instillation volume influences TBUT values [33]. Using 5.0 μl of 2.0% dye, Pflugfelder et al. [29] and Abelson et al. [34] have found TBUT values for normals in the range of 7 s, and have used breakpoints for classifying subjects as “dry” of ≤7 s. Moreover, Sullivan et al. [35] also using 5 μl of 2% sodium fluorescein, suggested a breakpoint of <7 s for a “dry” subject classification.

Another reason for the use of a TBUT breakpoint of ≤7.0 s was that it represented a conservative value for inclusion of dry subjects. Since this was a preliminary study, we endeavored to make certain that our dry breakpoint clearly differentiated normals from dry eye subjects, so that tear thickness differences, if present, would be observed.

We used a biomicroscope set at 16× and a yellow barrier filter to enhance observation of the initial dark spot (defined as the first change following a normal blink). The subject was instructed to blink normally three times, then to hold the eyelids open. Following the measurement, the eyelids were closed for 30 s, then the TBUT measured in the fellow eye. Each eye was measured three times in this alternating manner, and the mean value for each eye calculated.

Following TBUT determination, corneal staining was assessed using the Efron scale [23],modified with a five-sector corneal overlay [30,36], the bio-microscope set at 16× magnification, and the use of a yellow barrier filter. Corneal staining was graded using a 0–4 scale in 0.1 unit increments for five zones for each cornea. The worst sector score and the total staining score (sum of 0–4 scores for each of five sectors) for each eye were recorded, similar to that advocated by Appendix 5 of the DEWS Diagnostic subcommittee report [37]. Rose bengal conjunctival staining was assessed according to the NEI/Industry Workshop report (6 conjunctival zones), also using liquid dye [30] (3 μl of 1.0% non-preserved rose bengal instilled using a micropipette).

The criteria for determination of whether a subject had dry eye were as follows. At least 2 out of 3 criteria had to be met. Schein symptom questionnaire; dry if scored >7 of 24 maximum

• TBUT, average of three determinations; dry if ≤7 s since several studies have found normal TBUTs of less than 10 s [4,34,38-40];
• Fluorescein corneal staining, five zones; dry if ≥4 of 20 maximum (20%) OR rose bengal conjunctival staining, six zones (excludes cornea); dry is ≥4 of 24 maximum (17%) [30]

Two of the three criteria had to be satisfied (i.e., symptoms and staining, TBUT and staining, etc.) for the subject to be considered dry. Only one eye at each subject was used to collect tear thickness data, and always the same eye at both visits. The eye chosen was the eye that demonstrated the worst staining (0–4 scale), in any of the five sectors of either cornea [30]. Dry eye severity was based on the DEWS 2007 definition and classification subcommittee report [41]. The severity was assigned a Level 1–4, based on multiple signs and symptoms.

### Tear thickness measurement

**Figure 3:**
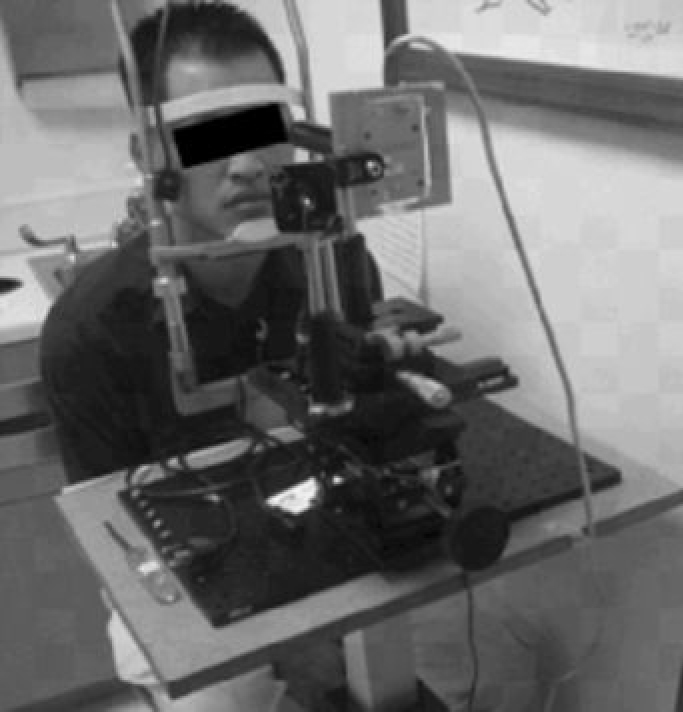
This figure displays the optical apparatus used for human tear film thickness measurements. The laser and the camera assembly were placed on mechanical stages equipped with X-Y-Z movement. The imaging system is mounted onto a track for scanning purposes. Subjects placed their head on the chin-rest and maintained a constant focal length with respect to the camera assembly. Data were streamed to a computer for further computational analysis.

The apparatus was mounted onto a table which was fastened to a headrest to stabilize the subject’s head (Figure 3). The subject was seated and instructed to fixate a target at approximately a 45 degree angle above horizontal. The laser and detector apparatus was aimed at the mid-peripheral inferior cornea for image capture. The subjects were instructed to blink normally three times to provide a consistent tear thickness, then the blink was suspended and images gathered for approximately 4 s.

### Thickness effect of a viscous eyedrop

To determine the length of time that a viscous artificial tear solution (Refresh Liquigel; Allergan, Irvine, CA) containing 1% carboxymethylcellulose as the primary viscolyzer might elevate tear film thickness, a 25 μl drop was instilled into the subject’s inferior fornix [42] using a positive displacement pipette. Tear thickness was monitored every 2 min post-instillation until 40 min post-instillation. For each measurement the subject blinked normally three times, and then held the eyelids open for approximately 4 s.

### Safety monitoring

Visual acuity was re-checked as one safety measure at ten minutes post-thickness measurement for subjects at Visit 1. The acuity could not be checked at Visit 2 since the drop was instilled immediately following the baseline thickness measurement. Visual acuity was assessed using a calibrated Snellen chart to monitor potential vision changes, with decrements greater than 2 letters considered significant. In addition, the inferior cornea in the region of the measurement was examined following the thickness measurement using slit-lamp biomicroscopy to assess cornea swelling or injury. Minimal laser injuries to the cornea have been defined as inducing a small white area involving the epithelium that develops within 10 min of exposure [16]. We examined each subject for the white spot, and for corneal staining post-thickness measurement following instillation of 2.0 μl of 1.0% non-preserved sodium fluorescein with use of a yellow barrier filter and the biomicroscope.

### Data analysis

The normal and dry eye tear thickness data were compared using 2-sample *t*-tests at Visits 1 and 2 of FASIC measurements. Intraclass correlation was used to examine repeatability, Visit 1 to Visit 2 for the normal and dry samples. We obtained the ICC from SPSS software (version 11.0; SPSS Inc.) using the one-way random model. We selected this model because we treated subjects as random and visit times as fixed. Correlational analysis of thickness relative to both Schein symptom scores (0–24 scale) and total corneal staining (0–20 scale) was undertaken. Preliminary tear thickness sensitivity and specificity analysis was undertaken using a receiver operating characteristic (ROC) approach [37]. A linear mixed model was used to compare the tear thickness over time for the retention of effect experiment at Visit 2. The subjects were treated as the random effect (as they were selected from a much larger population) and all treatments were fixed. Minitab software was used this analysis (version 15.0 from Minitab Inc.). Dunnetts Simultaneous Tests (corrects for multiple comparisons) were used to determine significant differences from the baseline, pre-instillation tear thickness.

## Results

### General results

The subject demographics for this study sample are summarized in Table 1. Forty subjects completed both visits, 22 dry eye subjects and 18 without dry eye. Of these, there were 19 males and 21 females, with more females in the dry group and all six of the soft lens wearers in the dry eye group. Overall, the ethnic groups are representative of the local population.

**Table 1:** Sample demographics and tear film data.

** **		**Sex**		**Ethnicity**				** **	** **	** **
**Age**		**Male**	**Female**	**Caucasian**	**Asian**	**Hispanic**	**Afri. Amer.**	**Schein score (0–24)**	**TBUT (s)**	**Corneal stain (0–20)**
Normal eyes (n=18)*		12	6	10	5	3	0	** **	** **	** **
Median	26.5	** **	** **	** **	** **	** **	** **	3.0	7.1	0.85
Mean	29.0	** **	** **	** **	** **	** **	** **	3.4	8.0	1.3
SD	8.3	** **	** **	** **	** **	** **	** **	1.8	3.2	1.5
Dry eyes (n=22)**		7	15	10	5	5	2	** **	** **	** **
Median	32.0	** **	** **	** **	** **	** **	** **	9.5	4.2	5.6
Mean	38.8	** **	** **	** **	** **	** **	** **	9.3	4.6	5.2
SD	13.8	** **	** **	** **	** **	** **	** **	4.0	2.5	2.2

*Did not satisfy more than one of the dry criteria (i.e., symptoms >7/24, TBUT ≤7.0 s, or corneal staining ≥4.0 on a 0–20 scale). **Severity designation is described in Methods and is assigned a level 1–4 based on the DEWS 2007 Definition and Classification report [41]. Thus, 3 subjects were Level 1, 12 Level 2, 7 Level 3, and none were Level 4.

Summary tear film data are also included in Table 1. Relative to dry eye severity, based on worst sector fluorescein staining, the 22 dry eye subjects fell into groups of 3 at trace, 14 who were mild, 5 moderate, and none in the severe category.

Regarding safety and comfort, the procedure was well tolerated in all subjects. There were no instances of visual acuity loss, visible corneal changes [16] or adnexal irritation. There were no occurrences of excess (i.e., beyond that present at the eligibility visit) corneal staining in the area of thickness measurement (using 2.0 μl of 1.0% NaFl, 10 min post-FASIC measurement).

### Tear film thickness in normals and dry eye subjects

**Figure 4:**
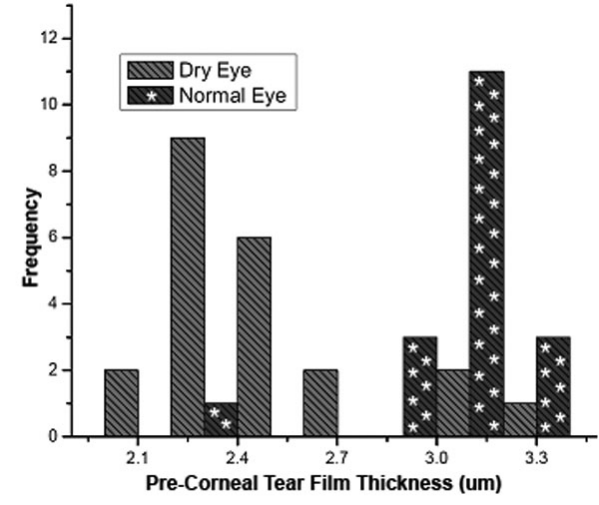
Human tear thickness data for normals (n=18) and dry eye subjects (n=22) at Visit 1.

We first considered whether the tear film thickness values were different for subjects with normal eyes compared to the dry eye group at visits 1 and 2 of FASIC measurements. For the first quantitative visit, the measured tear film thickness values averaged 3.05±0.21 μm and 2.48±0.27 μm for normals (n=18) and dry eye subjects (n=22), respectively. These thicknesses were significantly different (2-sample *t*-test, p<0.001). At the second (repeatability) visit, mean tear thickness values were 3.06±0.18 μm and 2.46±0.25 μm for normals (n=18) and dry eye subjects (n=22), respectively, and were also significantly different (two-sample *t*-test, p<0.001). Figure 4 shows the histogram of the individual tear film thickness values.

**Figure 5:**
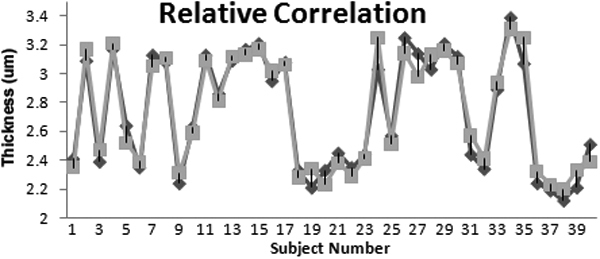
Relative correlation between the two FASIC visits is shown.

The repeatability of the thickness values in normal and dry eye subjects for the two visits was evaluated by intraclass correlation coefficient (ICC). ICC values of 0.935 and 0.950 were obtained for the normal and dry eye groups, respectively. These results demonstrate good repeatability of this technique. Figure 5 displays the relative correlation between the data from the two visits with this imaging platform.

**Figure 6:**
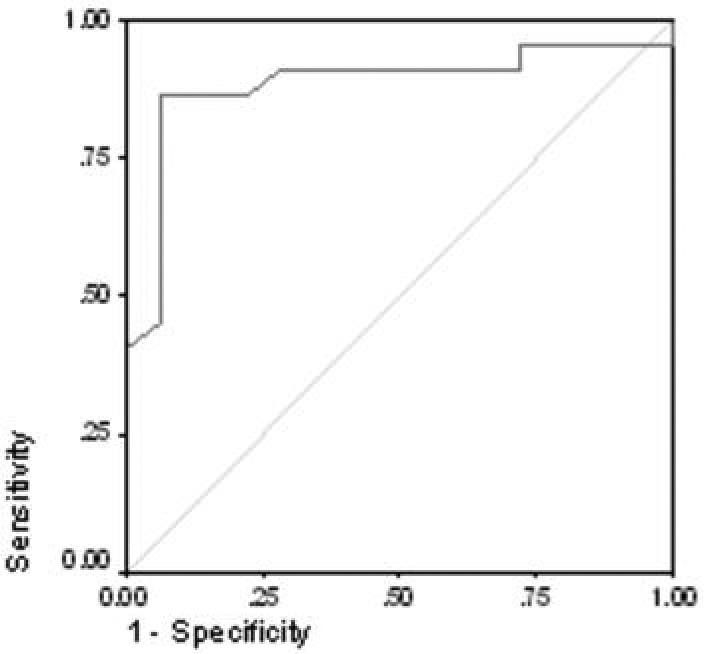
Receiver operating characteristic curve (ROC) for tear thickness using data from Visit 1. The AUC is 0.886. At a cut-point for tear thickness of ≤2.75 μm, to diagnose dry eye, the test sensitivity is 0.86, and the specificity is 0.94.

For a diagnostic method, the Dry Eye Workshop has recommended assessing the test sensitivity (ability to detect disease) and specificity (the ability to correctly identify those without disease) for measures of dry eye [37]. Since we diagnosed dry eye disease using conventional tests, tear thickness could be analyzed for these parameters. We plotted sensitivity against 1–specificity to generate a receiver operating characteristic (ROC) curve as shown in Figure 6. Inspection of the ROC curve provided an ACU of 0.886 and suggested an optimal cut point for tear thickness of 2.75 μm. The corresponding sensitivity and specificity were 0.864 and 0.944, respectively.

Correlational analysis was undertaken at both visits to compare tear thickness against total corneal staining score (0–20 scale), Schein symptom score (0–24 scale) and tear stability (TBUT). For total staining, moderate correlation was found (drys only; Pearson’s R of −0.574 (p=0.005) and −0.424 (p=0.049) for Visits 1 and 2, respectively. No relationship was found for tear thickness and Schein score (drys only; Pearson’s R=0.120 [p=0.594] and R=0.033 [p=0.885] for Visits 1 and 2, respectively). For tear stability, we found slight negative correlations between TBUT and tear thickness for normals at both visits, and slight positive correlations for dry eyes, but none of the relationships was statistically significant. However, correlational analysis using all subjects (n=40) for tear thickness versus tear stability (TBUT) found a moderate positive correlation (Pearson’s values: R=0.419 and 0.412 for visits 1 and 2, respectively) that were highly statistically significant (p=0.007 and p=0.008 for visits 1 and 2, respectively). This suggests that even with a modest overall sample size that as tear stability increases, so does tear thickness, as might be expected.

### Thickness effect of a viscous eyedrop

The effect on tear thickness of a viscous topical formulation was examined in the dry eye subject sample (n=19). Following a single 25 µl drop instillation into the inferior fornix, tear thickness was monitored every 2 min until 40 min post-instillation.

**Figure 7:**
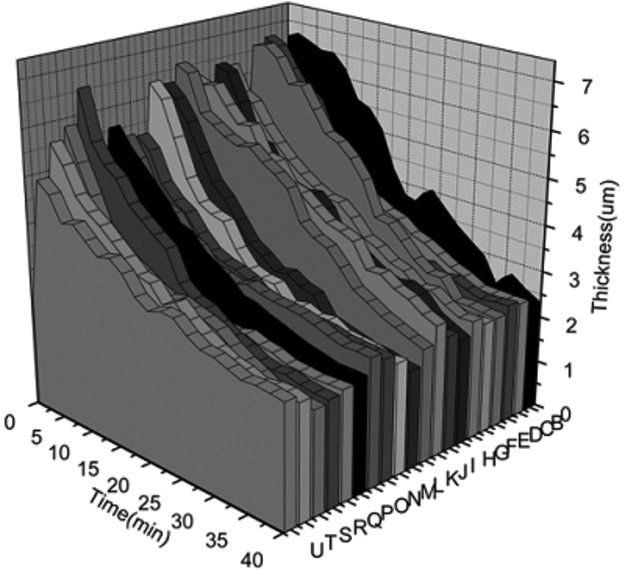
This plot represents the changes in tear film thickness by subject as a function of time upon instillation of Refresh Liquigel (Allergan, Irvine, CA). These data were used to calculate the mean retention of effect time for the sample, 32 min, to become statistically indistinguishable from the mean baseline tear thickness.

Figure 7 displays the retention of effect data obtained. The phrase “retention of effect” is used since we measured the length of time that a beneficial effect (i.e., a thickness increase) was evident, not the direct residence time of the drop. The mean baseline thickness was 2.49±0.31 µm. Tear film thickness increased to a mean value of 5.95±0.68 µm at measurement number 1 with the maximum and minimum value of 6.85 µm and 4.62 µm, respectively. Tear film thickness decreased over time and recovered to baseline levels in 16 of the subjects. Tear thickness became less than the baseline value in 1 of the subjects in this study. Tear thickness was statistically significantly different (greater) than baseline until 32 min post-instillation (Dunnett’s simultaneous tests; p=0.047 at 32 min compared baseline).

**Figure 8:**
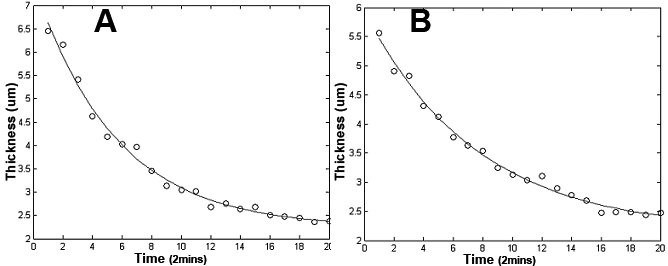
Individual data sets from every subject have been fitted with the single exponential function. **A**, **B**: Show two typical data sets along with their single exponential fittings. Decay constant was calculated for every subject and an average value (±STD) 9.52±2.02 of was obtained.

The retention time data were fitted with a single exponential function using the method of least-squares to find the decay time in each data set. Figure 8 shows the averaged data for each time point fitted with a single exponential function. This set of data had a time-constant value of 10.71±4.05 min and an amplitude value of 5.03±1.54. As can be seen, the data exhibits a true exponential behavior.

## Discussion

We investigated the ability of a recently described [15], non-invasive method, based on fluctuation analysis using spatial image correlation, to estimate the thickness of the human tear film. The method requires relatively inexpensive equipment and appears safe for multiple measurements of the tear film since we observed no corneal distress or change in visual acuity following the procedure. The mean values found for non-dry eye subjects were approximately 3.1 um (±0.2) for both visits (n=18). These are in good agreement with the data of King Smith and coworkers [9,13] who found a mean value of approximately 3.0 µm in normal humans, also using a direct, non-invasive method. Moreover, at both visits we found a statistically significant difference in tear thickness (p<0.001 at visit 1 and 2) between dry eye subjects (~2.5 µm, n=22) compared to non-dry eye individuals. For both non-dry eye subjects and dry eye subjects the short-term (within one week) repeatability was very high (ICC of 0.94 and 0.95, respectively).

The thickness comparison data and the repeatability data for the two subject groups demonstrated highly significant differences due to the sensitivity and specificity of this quantitative imaging technique.

We explored the diagnostic test efficacy of tear thickness measurement using the FASIC approach to differentiate non-dry eyes from those with dry eye using the approach suggested by the recent DEWS report [37]. We used standard tests (i.e., the presence or absence of irritation symptoms, fluorescein tear stability, corneal and conjunctival staining) to classify subjects as dry or non-dry, then examined the sensitivity of tear thickness to characterize these subjects as dry or non-dry. The sensitivity and specificity data used to generate the ROC curve (Figure 6) suggest that thickness can indicate dry eye at a cut-point of thickness ≤2.75 µm with a sensitivity of 86% and a specificity of 94%. These test efficacy numbers are quite promising, although they need to be verified in an independent sample of normal and dry eye subjects [37].

The goal of topical therapy in dry eye is to relieve symptoms in conjunction with potentially replacing deficient components (e.g, aqueous fluid) so that the tear film is thickened and stabilized in the short-term to provide healing of the ocular surface over time. We undertook a brief study of the effect of a viscous topical drop and examined the thickness changes occurring and the length of time that the thickness effect could be observed. This latter phenomenon is referred to as the retention of effect rather than a direct estimate of residence, or ocular surface dwell time.

Our results suggest that, compared to the baseline tear thickness in the dry eye group (mean=2.48±0.32 μm), the tear film becomes significantly thicker at 2 min post-instillation (mean=5.95±0.68 μm) and remains significantly thicker (p<0.05) until 32 min post-instillation. This retention of effect time is in agreement with the direct residence time reported earlier (approximately 41 min [43]) for the same artificial tear. Moreover, the time of approximately 32 min of beneficial effect is similar to that found for non-invasive tear stability when a thickened artificial tear was examined in dry eye subjects [3].

### Future rsearch

This work was a preliminary study that provided initial tear film thickness data. However several issues remain that require further consideration. One concern is the confounding effect of subject age, sex, and prior contact lens wear. While age is expected to influence tear thickness, the potential differences due to sex and contact lens wear require studies with a larger number of subjects. Other potentially important issues are the severity of the dry eye condition, and the sub-type of dry eye (i.e., meibomian gland dysfunction compared to aqueous tear deficiency). Similarly, a fundamental question is whether various dry eye etiologies result in differing tear film thicknesses.

Given the relatively small bulk of the apparatus used in this study, future research could investigate tear thickness in other areas of the cornea including: central, superior, nasal, temporal, and inferior regions. The scanning can be done simply by moving the imaging system along the ocular surface.

Another application of the methodology could be in drug delivery. Drug delivery vehicles could be evaluated for retention of effect, to determine whether a given formulation provides efficacy in delivering medication into the anterior chamber. Healthy normals should be evaluated as controls, as well as diseased subjects, since patients with intraocular conditions may exhibit normal external ocular health. Healthy normals could also participate in future studies to explore the biophysics characteristics of tear film. In this technique, a low power 635 nm laser was used and we did not detect obvious reflex tearing or other untoward subject behavior. While no adverse effects were detected it is possible that the intensity of the light could have physiologic effects on subjects such as increased blink rate and increased tearing. Future studies need to evaluate this possibility along with assessing the application of a longer wavelength (i.e., in the infrared range) light source that may produce fewer physiologic effects.

### Conclusions

In this work the thickness of a human pre-corneal tear film was quantified. The residence time of a lubricating eye drop (Refresh Liquigel; Allergan, Irvine, CA) was also studied on subjects with dry eye. Our measurements revealed the details of the changes in thickness as a function of time.

The FASIC technique appears to provide valid human tear thickness values and expected thinner values in dry eye. It is repeatable in both normals and dry eye subjects over a short time frame, and appears to provide good test efficacy when used as a diagnostic test for dry eye. Moreover, tear thickness appears to be an indicator of topical formulation retention of effect by being able to monitor thickness changes over time. As such it could be used to monitor both the short-term efficacy of dry eye treatment preparations and to determine whether tear thickness returns to normal following longer-term treatment.
